# Cancer Research UK procedures in manufacture and toxicology of radiotracers intended for Pre-phase I positron emission tomography studies in cancer patients

**DOI:** 10.1038/sj.bjc.6600212

**Published:** 2002-04-08

**Authors:** E O Aboagye, S K Luthra, F Brady, K Poole, H Anderson, T Jones, A Boobis, S S Burtles, P Price

**Affiliations:** Cancer Research UK, PET Oncology group, Imperial College of Science, Technology and Medicine, Faculty of Medicine, Department of Cancer Medicine, Hammersmith Campus, Rm. 242 MRC Cyclotron Building, Du Cane Road, London W12 0NN, UK; MRC Cyclotron Unit, Department of Radiochemistry, Hammersmith Campus, Du Cane Road, London W12 0NN, UK; Imperial College of Science, Technology and Medicine, Faculty of Medicine, Department of Clinical Pharmacology, Hammersmith Campus, Du Cane Road, London W12 0NN, UK; Cancer Research UK, 10 Cambridge Terrace, London NW1 4JL, UK

**Keywords:** Pre-phase I, toxicology, positron emission tomography (PET), radiopharmaceutical

## Abstract

Radiolabelled compounds formulated for injection (radiopharmaceuticals), are increasingly being employed in drug development studies. These can be used in tracer amounts for either pharmacokinetic or pharmacodynamic studies. Such radiotracer studies can also be carried out early in man, even prior to conventional Phase I clinical testing. The aim of this document is to describe procedures for production and safety testing of oncology radiotracers developed for imaging by positron emission tomography in cancer patients. We propose strategies for overcoming the inability to produce compounds in sufficient quantities via the radiosynthetic routes for full chemical characterisation and toxicology testing including (i) independent confirmation as far as possible that the stable compound associated with the radiopharmaceutical is identical to the non-labelled compound, (ii) animal toxicity studies with ⩾10 times (typically 100 times) the intended tracer dose in humans scaled by body surface area, and (iii) patient monitoring during the radiotracer positron emission tomography clinical trial.

*British Journal of Cancer* (2002) **86**, 1052–1056. DOI: 10.1038/sj/bjc/6600212 www.bjcancer.com

© 2002 Cancer Research UK

## 

New drug discovery methods such as computer-aided drug design and combinatorial chemistry with high-throughput screening are producing a large number of anticancer agents for *in vivo* evaluation. Due to the limited resources available for developing all these drug candidates, there is a need to efficiently integrate pharmacokinetic and pharmacodynamic studies into pre-clinical and early clinical testing. Early information on tumour and normal tissue pharmacology of new agents, as well as response of tumours to therapy with such agents will aid the rapid and rational translation of ideas from the laboratory to the clinic. Imaging with positron emission tomography (PET) can offer the opportunity to study pharmacokinetics and pharmacodynamics non-invasively in patients. PET studies involve the administration of compounds labelled with positron-emitting isotopes (radiotracers), which have been formulated for intravenous injection (radiopharmaceuticals). Generally, these radiopharmaceuticals are produced at high specific radioactivity, i.e. a high level of radioactivity is associated with a very small amount of stable compound or carrier thus allowing administration and detection of tracer doses of the material in patients. Despite the opportunities provided by PET (Table 1

**Table 1:**
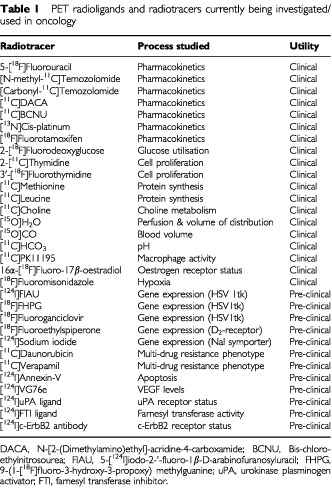
PET radioligands and radiotracers currently being investigated/used in oncology

), there are currently no published guidelines on the safety testing of radiopharmaceuticals prior to use in clinical trials in patients with cancer. The primary aim of this document, therefore, is using Cancer Research UK's experience of this technique to propose minimum requirements for product quality, animal toxicology and initial clinical studies at tracer doses of PET radiopharmaceuticals for oncology, which are consistent with patient safety. In preparing this document, existing guidelines on related subjects were taken into consideration (Begent *et al*, 1986, 1993; Burtles *et al*, 1995; FDA, 1998; CRC/EORTC, 1999). Despite the general considerations discussed here, each compound should be reviewed by local, national and/or international bodies involved in medicine control and radioactive substance administration.

### Scope of this document

This document relates to studies on cancer patients only and does not apply to studies on healthy volunteers. The procedures described in this study apply to ‘tracer dose’ studies of novel agents in cancer patients, which may be performed prior to conventional Phase I trials. Tracer dose studies may include (i) pharmacokinetic evaluation of new anticancer agents in humans by PET, and (ii) evaluation of new PET radiotracers designed for investigating the physiology of human tumours or providing pharmacodynamic/mechanistic end points.

### Background and experience to date

Pre-phase I studies of anticancer agents will enable tumour and normal tissue pharmacokinetic information to be obtained prior to Phase I clinical trials. Translational research questions that justify a Pre-phase I study to be performed include, whether the novel agent distributes to the tumour, to what extent the agent distributes to normal tissues and whether the metabolite profiles in rodents and humans are similar. This is particularly important for novel agents that are designed to be tumour-specific. Opportunities also exist for studying pharmacokinetic modulation and scheduling. Regarding the safety of administering tracer doses of anticancer agents, administered radiopharmaceuticals are likely to contain 100–1000 times less drug than the starting dose for Phase I studies in patients (Saleem *et al*, 2001a). The risk to patient volunteers is, therefore, considered to be minimal. In addition, the amount of radioactivity administered to patients at any one time is generally not greater than 1–3 years of annual background radiation exposure within the UK. Cancer patients, most of them with incurable disease, will be likely to receive cytotoxic drugs or radiation as part of their management, which are genotoxic. It is therefore normal practice not to consider genotoxicity as a risk to the patient in such early protocols.

Under the auspices of Cancer Research UK, studies with carbon-11 radiolabelled N-[2-(dimethylamino) ethyl]acridine-4-carboxamide [^11^C]DACA (Harte *et al*, 1996; Osman *et al*, 1997; Saleem *et al*, 2001a), a topoisomerase inhibitor, established the feasibility of performing PET studies with radiolabelled anticancer agents at tracer doses prior to the commencement of Phase I clinical trials. Such studies were possible because of the ability to produce radiopharmaceuticals with high specific radioactivities associated with extremely small amounts (μg) of stable compound, and the inherent sensitivity of PET scanning. Since pre-clinical toxicology data and the proposed Phase I starting dose of DACA were available, PET studies could be carried out using formulations of [^11^C]DACA containing less than 1 out of 1000 of the proposed Phase I starting dose of DACA (18 mg m^2^). This allowed studies of [^11^C]DACA to be performed in man 1 year prior to the Phase I trial (Harte *et al*, 1996; Osman *et al*, 1997; Saleem *et al*, 2001a). In addition to drug distribution studies, metabolism, and in some cases, mode of action of new compounds can be studied in man prior to the Phase I trial. For instance, selective labelling of the methylating agent temozolomide in the carbonyl and methyl positions has enabled demonstration of the ring-opening of this compound in man (Saleem *et al*, 2001b). Although these latter mechanistic studies followed Phase I studies of temozolomide, it demonstrates the potential of incorporating such strategies into early clinical development.

### Radiochemistry

Positron emitting radioisotopes, such as, carbon-11 (half-life, 20.4 min) and fluorine-18 (half-life, 109.8 min) are produced using a cyclotron. These radioisotopes are obtained from the cyclotron in simple chemical forms. Carbon-11 is obtained as either [^11^C]carbon dioxide or [^11^C]methane and fluorine-18 is obtained as either [^18^F]fluoride or [^18^F]fluorine. Radiochemistry is performed on these simple molecules in order to incorporate them into the compounds of clinical interest. This generally involves several steps including conversion of the radioisotope into a reactive intermediate, which is then used to radiolabel a suitable precursor giving the radiolabelled compound of interest. The radiolabelled compound is then isolated using high performance liquid chromatography (HPLC). After removal of HPLC mobile phase, the residue is formulated and dispensed for injection under an aseptic environment.

Compounds radiolabelled with carbon-11 or fluorine-18 (via fluoride only) are produced at high specific radioactivity, i.e. a high level of radioactivity is associated with a very small amount of the stable compound or ‘carrier’. Although such radiolabelled compounds are said to be ‘no carrier added’ (NCA), a sufficient amount of stable compound is usually present in the radiopharmaceutical after radioactive decay to allow one to obtain a mass spectrum of the compound. For carbon-11 chemistry, the position of incorporation of the radiolabel can be verified by carrying out co-labelling experiments in which very small amounts of carbon-13 labelled reagents are mixed with the carbon-11 equivalents and used in the radiosynthesis. The purified product is then examined by carbon-13 nuclear magnetic resonance and mass spectrometry.

Generally, synthetic strategies are designed to produce a radiolabelled compound that is chemically identical to the compound of clinical interest. The radiolabelled compound is purified chromatographically to eliminate radiochemical and chemical by-products of the synthesis. Unlike other non-isotopic radiolabelling strategies, e.g., labelling of antibodies, compounds radiolabelled with carbon-11 are chemically identical to the non-radioactive compound. When compounds contain stable fluorine then the fluorine-18-labelled version is also chemically identical, e.g. 5-[^18^F]fluorouracil. Given the constraints of the short half-lives of the radioisotopes and the limited range of labelling agents that can be produced, it is usually not possible to use the same synthetic route that is used in the synthesis of the non-radioactive compound for which toxicity data would have been obtained.

### Production and quality control

In most centres, radiopharmaceuticals are produced for clinical PET studies using documented Standard Operating Procedures (SOPS). The radiosyntheses should be performed according to Good Manufacturing Practice (GMP) in approved production facilities, e.g. within the UK, facilities with a Medicines Control Agency (MCA) ‘Specials’ License for the preparation of radiopharmaceuticals (MCA, 1997). Equivalent regulatory bodies in other countries such as the US Food and Drug Administration (FDA) in North America are responsible for issuing guidance notes and regulations. Due to the short half-life of the positron emitters, fewer Quality Control tests can be carried out on radiopharmaceuticals before their release for clinical studies. Thus, the continuous assessment of the effectiveness of the Quality Assurance system before initiation of the Pre-phase study is very important. This requires compliance with the relevant rules and guidance for pharmaceutical manufacture and distribution. In accordance with the UK MCA guidance, for instance, dispensing of radiopharmaceuticals should be carried out in Class I isolators and a record of trained personnel who perform various activities should be kept to comply with GMP. Production of different radioactive products in the same work stations (‘hot cells’) and at the same time should be avoided in order to minimise the risk of cross-contamination or mix-up. Process validation, in-process controls and monitoring of process parameters and environment assume particular importance in cases where it is necessary to take decisions to release or reject a batch or product before all tests are completed (MCA, 1997). Formulated samples (usually for intravenous administration) are subjected to in-house independent Quality Control procedures and must meet defined specifications, which include chemical and radiochemical purities with set limits on stable compound and impurities. These limits are set by production chemists, Quality Control team, biologists and clinical investigators based on radiosynthesis, analytical methodology, limit of detection of the stable compound and impurities, purity profile, nature of impurities, effect of stable compound on tracer kinetics and study type. These limits should be reviewed as new data become available. In addition, analysis of residual organic solvents, e.g., acetonitrile, in the radiopharmaceutical should be carried out and must meet defined specifications (EMEA, 1998). Furthermore, steps must be taken to ensure that samples are sterile, isotonic, apyrogenic and have a suitable pH. Currently, a monograph for PET oncology radiopharmaceuticals is only available for 2-[^18^F]fluorodeoxyglucose (EP, 1996). Examples of radiochemical and chemical purities of radiopharmaceuticals for PET oncology recently analysed by the independent Quality Control team within the authors' own laboratories are shown in Table 2

**Table 2:**
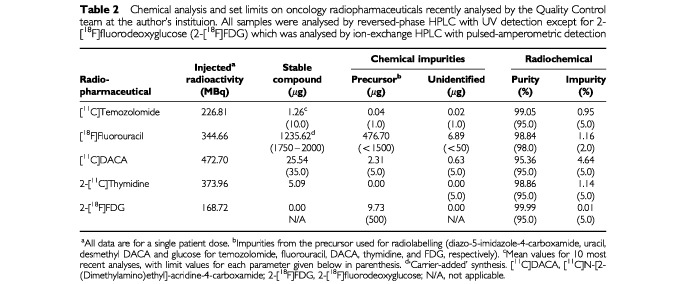
Chemical analysis and set limits on oncology radiopharmaceuticals recently analysed by the Quality Control team at the author's instituion. All samples were analysed by reversed-phase HPLC with UV detection except for 2-[^18^F]fluorodeoxyglucose (2-[^18^F]FDG) which was analysed by ion-exchange HPLC with pulsed-amperometric detection

. Radiopharmaceuticals having very high specific radioactivity often undergo decomposition due to radiolysis because of the high specific activity *per se* and the need to start the synthesis with very high quality radioactivity. It is, therefore, recommended practice to evaluate the stability of the radiopharmaceutical during test runs for up to 4 h (for carbon-11 and fluorine-18) after radiosynthesis, to assure that no radiochemical or chemical changes have occurred.

### Toxicology

Since Pre-phase-1 studies may involve the first time administration of a compound to humans, there is the need to establish general procedures for pre-clinical toxicology testing of these radiopharmaceuticals, despite the very low dose of drug required for detection by ‘tracer’ PET. Given the low levels of radiolabelled compound required and the limited number of doses each patient will receive (typically 1), it is recommended that single-dose toxicology studies of the radiopharmaceutical in the formulation to be used in the tracer studies in man are performed. If multiple dose studies are carried out, the radiopharmaceutical is administered weeks apart and, from a toxicological stand-point, is, therefore, considered equivalent to a single dose. Due to differences in synthetic routes, impurity profiles and occasionally, vehicle used in formulation of radiopharmaceuticals, the toxicology studies should be done even if other (non-radiopharmaceutical) toxicology data are available. As the trials are to be carried out in cancer patients with limited life expectancy, long-term repeat dose toxicology, genotoxicity and carcinogenicity studies may usually be omitted, but should be considered on a case by case basis. Biodistribution studies after a single injection of the radiopharmaceutical should be performed to determine the dosimetry. With regards to impurities generated during the radiosynthesis, it should be emphasised that the parent compound will be given at only tracer doses, and hence exposure to minor impurities would be negligible. They should, however, still be considered and limits set. Due consideration should also be given to levels of the other components in the formulation, such as anti-oxidants and residual solvents. Also of importance is the issue of immunogenicity. If PET radiopharmaceuticals are to be given more than once to the same patient then there is the theoretical risk of hypersensitivity. As with normal pharmaceuticals, this issue should be addressed by inspecting the structures of compounds for any possible alerts for immunogenicity by analogy with related compounds, e.g., peptides in general or the presence of a penicilloic acid moiety. Expert advice should be obtained if potentially immunogenic compounds are encountered. In addition to animal toxicology, patients should be monitored for toxicity as with standard Phase I studies, and the patient studies should be performed in accordance with the guidelines for Good Clinical Practice (GCP) (ICH, 1996).

## GENERIC GUIDELINES FOR CANCER INVESTIGATIONAL AGENTS

Following a meeting of a Cancer Research UK expert committee on PET Pre-phase I trials, a number of recommended procedures were developed. These recommendations will be reviewed in the next 5-years as more data become available. It is recommended that the following studies should be performed for candidate compounds: (1) Chemical identity and purity, (2) Animal toxicology, (3) Clinical trial.

### (1) Chemical identity and purity

The major requirement should be to provide evidence that the radiopharmaceutical prepared via the radiosynthetic route is identical to the non-radiolabelled reference compound. If the radiosynthetic route differs from that for the non-radiolabelled compound then the radiosynthetic route should be documented and be available for review by an independent body. The chemical identity of the radiolabelled compound after decay should be determined by mass spectrometry and nuclear magnetic resonance methods if feasible. Furthermore, radiochemical purity, radionuclidic purity, and chemical purity of the radiopharmaceutical should be determined and documented. Radiochemical and chemical impurities may be identified by HPLC-UV and HPLC-mass spectrometry methods. In general, the procedure used will depend on the chemical structure and radiosynthesis used to produce the compound. Once a radiosynthesis has been developed, it is normal practice to perform a number of runs (3–5) to demonstrate the robustness and reproducibility of the manufacturing process and prove that the radiopharmaceutical is pure, sterile, isotonic, apyrogenic, stable and of suitable pH. The trial runs also ensure that the total radioactivity, specific radioactivity and radiochemical purity are appropriate for the clinical study. Batch manufacturing records of the radiosynthesis and quality control analyses should be documented and be available for inspection. Since the radiosynthetic route may differ from that for the non-radiolabelled compound, the product may contain impurities, which have not been subjected to safety evaluation. Before any material is made for clinical use, limits on the amounts of identified and unidentified impurities should be set on a case by case basis. A standard operating procedure (SOP) should be prepared for production of future batches. This must be done before the radiopharmaceutical can be administered to man. Due to the difficulty in performing extensive tests on each patient dose (batch) produced, it is not usual practice to obtain a certificate of analysis. Instead, a formal recorded decision must be taken by the Qualified Person (Quality Control officer) on the conformity of the batch to ensure that they meet the pre-defined standards specified in the SOP.

### (2) Animal toxicology

Toxicology and biodistribution studies are designed to ensure that the radiopharmaceutical is non-toxic from a chemical and radiation viewpoint at the doses to be administered to patients. Any previous animal toxicology data on the compound should be documented and used in designing the Pre-phase I study. The studies should be performed under the highest quality standards possible (equivalent to GLP). The following recommendations should be adhered to:

#### Investigator

The principal investigator should have expertise in pre-clinical toxicology testing. Alternatively, the toxicology work may be subcontracted to a certified commercial establishment.

#### Protocol

A protocol for the toxicology studies should be prepared and documented.

#### Chemical toxicity

The radiopharmaceutical (after radioactive decay) formulated in the recommended vehicle should be used. The radiopharmaceutical should be administered as a single dose equal to at least 10 times (and typically 100 times) the intended tracer dose in humans (scaled by body surface area). The intended tracer dose in humans is the total amount (in μg) of the stable compound (associated with the desired level of radioactivity in MBq) that will be administered to a patient. The total patient dose is usually of the order of 1–30 μg for ‘non-carrier added synthesis’ (Table 2). Thus, the dose is selected based on the achievable radiosynthetic yield. The recommended route for clinical administration (usually intravenous) should be used. Aliquots of dosing solutions should be analysed for drug content by HPLC-UV, gas chromatography-mass spectrometry, liquid chromatography-mass spectrometry or other sensitive analytical methods. Toxicology studies in one species is adequate (mouse or rat; male unless there is a reason to use females). Three to five animals should be treated with vehicle only (controls) and 3–5 animals should be treated with the drug. Animals should be monitored for 14 days for death, changes in body weight, and clinical signs (e.g., neurological, gastrointestinal, performance status, hair characteristics, feeding status, lacrymation, and urine colouration). Animals should be killed on day 14 after the above examinations and macroscopic pathology evaluated. If possible, clinical chemistry and haematology analyses should be carried out, but histopathology, genotoxicity and carcinogenicity studies are not required unless clearly indicated. Reproductive toxicology studies are not required as long as patients of reproductive age will be advised to take appropriate precautions to avoid pregnancy, and pregnant and lactating patients will be excluded from any clinical studies. A full toxicology study (maximum tolerated/administrable dose endpoint; 7-day repeat) would be required if significant weight loss ensues or if any of the observational endpoints mentioned above are encountered. A full toxicology study would also be required if the 10-100X tracer dose for the non-radioactive compound used in the toxicology studies is greater than 10% of the maximum tolerated dose (if this is known) or the effective therapeutic dose (if the MTD is not known) in the same species.

#### Radiation toxicity

Dosimetry studies should be performed with the radiopharmaceutical in rodents (usually adult male/female rats) over several time points depending on the biological and physical half-lives of the radioisotope. The radioactivity in blood, urine and tissues (gonads, bone marrow, colon, lung, stomach, bladder, breast, liver, oesophagus, thyroid, skin, bone surface, adrenals, brain, upper large intestine, small intestine, kidney, muscle, pancreas, spleen, thymus, uterus, heart and eye), expressed as a percentage of injected radioactivity should be determined to enable calculation of the effective radiation dose (mSv) to each tissue. In the UK, the effective dose is required by the Administration of Radioactive Substances Advisory Committee (ARSAC).

### (3) Clinical trial

As for all clinical trials, the details of the objectives, patient eligibility, treatment schedules, endpoints and all tests should be documented in the clinical trial protocol. This, along with the patient information-sheet must be submitted to the local hospital research ethics committee (LREC). Regulatory approval for the trial must be obtained from the appropriate Regulatory Authority. In the UK, this is the Medicines Control Agency and for academic studies an application for a Doctor and Dentist's Exemption (DDX) would be made, although this is likely to change with the implementation of the European Clinical Trials Directive. The protocol should define all the tests to be performed on the patients. Although patients will only receive a tracer dose of drug and no toxicity is expected, all patients must be monitored for safety (side effects) for up to 24 h after dosing. Tests should include ECG, blood pressure, heart rate, clinical chemistry and urine analysis. The protocol will define the eligibility criteria for participation in the trial. As for all trials of novel agents, pregnant and lactating women should be excluded and patients of reproductive age should use adequate contraception for the duration of the study.

#### ARSAC

Within the UK, a certificate for the administration of radioactive medicinal products under the Medicines Act 1968 and the Medicines (Administration of Radioactive Substances) Regulations 1978 must be obtained by the principal investigator from ARSAC prior to patient studies (ARSAC, 1998). This is a certificate of limited validity that allows specific radiolabelled compounds of pre-defined effective dose to be administered to cancer patients (also for normal volunteers and other conditions).

In summary, Pre-phase I studies will enable pharmacokinetic and pharmacodynamic studies to be carried out early in the clinical development of an anticancer agent providing added value to the development of the drug. The procedures proposed describe minimal toxicology studies consistent with safe use to enable PET studies to be carried out safely and in a timely fashion. The key components introduced in this document are the proposed strategies for overcoming the inability to produce compounds in sufficient quantities via the radiosynthetic routes for full chemical characterisation and toxicology testing. Specifically, these are:

- to independently confirm as far as possible that the stable compound associated with the radiopharmaceutical is identical to the non-radiolabelled compound.
- to perform animal toxicity studies at ⩾10 times (typically 100 times) the intended tracer dose in humans (scaled to body surface area).
- to undertake careful monitoring of patients during the tracer PET studies.

The proposed procedures are based on strategies given earliear.
